# Numerical and Experiment Studies of Different Path Planning Methods on Mechanical Properties of Composite Components

**DOI:** 10.3390/ma14206100

**Published:** 2021-10-15

**Authors:** Dongli Wang, Jun Xiao, Xiangwen Ju, Mingyue Dou, Liang Li, Xianfeng Wang

**Affiliations:** 1 College of Material Science and Technology, Nanjing University of Aeronautics and Astronautics, No. 29, Yudao Street, Qinhuai District, Nanjing 210016, China; wdl@nuaa.edu.cn (D.W.); j.xiao@nuaa.edu.cn (J.X.); xiangwen_ju@nuaa.edu.cn (X.J.); 2 Nanjing Chenguang Group, No.1, Zhengxue Street, Qinhuai District, Nanjing 210016, China; Luna20211011@163.com (M.D.); liang1757761210@163.com (L.L.)

**Keywords:** advanced composite, automatic fiber placement, path planning, multi-objective optimization, mechanical properties

## Abstract

The purpose of this paper is to study the effects of different trajectory planning methods on the mechanical properties of components. The scope of the research includes finite element simulation calculation and experimental tests of the actual structure. The test shall be carried out in the whole load range until the failure of the structure occurs. Taking the composite conical shell as an example, a variable angle initial path generation method of the conical shell surface is proposed, and the parallel offset algorithms based on partition and the circumferential averaging are proposed to fill the surface. Then, finite element analysis is carried out for the paths that satisfy the manufacturability requirements, the analysis results show that the maximum deformation and maximum transverse as well as longitudinal stress of fiber of circumferential averaging variable angle path conical shell are reduced by 16.3%, 5.85%, and 19.76%, respectively, of that of the partition variable angle path. Finally, the strength analysis of conical shells manufactured by different trajectory design schemes is carried out through finite element analysis and actual failure tests. The finite element analysis results are in good agreement with the experimental results of the actual structure. The results show that the circumferential uniform variable angle has good quality, and it is proved that the path planning algorithm that coordinates path planning and defect suppression plays an important role in optimizing placement trajectory and improving mechanical properties of parts.

## 1. Introduction

Fiber reinforcement composites have been widely used in aerospace and other high-tech fields due to their light weight, high strength, high modulus, fatigue resistance, corrosion resistance, strong designability, and simplicity to realize automatically integrated manufacturing [1]. Automatic fiber placement (AFP) is one of the most important technologies for manufacturing carbon fiber reinforced plastic (CFRP) components, which can realize the manufacturing of large-size and complex composite structural parts because each slit-tape is driven individually [2,3,4]. Moreover, it is the development direction of low-cost manufacturing technology of composite materials.

AFP is a manufacturing process in which a numerically controlled robot or CNC machine delivers prepreg tows of thermoset composites at a specific position and orientation through a placement head. It can lay as many as 32 fiber tows simultaneously at a constant angle which is often set as 0°, ±45°, or 90°, and the width of a prepreg tow is usually within the range from 3.175 mm up to 25.4 mm. These tows are transported from a creel system through the placement head and deposited onto the mold surface or already existing plies along a specific path [5,6]. Path planning is an extremely important process in AFP [7], which directly determines the quality of the manufactured component and the efficiency of the placement.

Over the past decades, studies on AFP-based path planning were focused on initial path design and optimization [1,8,9,10,11], generation of offset path [10,11], and quality control [12,13,14]. Moreover, models of path deformation were also established to obtain the relationship between placement parameters and layup quality of fiber path [1,14]. Many studies for path generation for open-contoured structures were carried out by Shirinzadeh, Long Yan et al. To sum up, the main construction methods of the initial reference path mainly include [15,16,17,18]: forming a fixed angle with an axis [10], along the direction of principal stress distribution or ply bearing information [19], surface–plane intersection strategy [20], projecting the reference line on the mold surface [21], etc. Shirinzadeh et al. [11] proposed a novel path planning algorithm for open-contoured structures, the initial reference path is generated by projecting the major axis on the mold surface, the entire surface is then completely covered by continuously offsetting the initial reference path. However, the placement direction of the parallel path is severely deflected and defects like wrinkles may occur since the steering radius is not controlled, the algorithm is only suitable for simple surfaces. A new path planning algorithm, which uses a surface–plane intersection strategy to construct the initial path, is proposed in [7]. For complex and closed profile parts, the traditional path planning algorithm of isometric offset in fiber placement is difficult to meet both angle deviation and curvature requirements, and it is very easy to cause the angle deviation to exceed the limit. To overcome this limit, Duan [9] proposed an angle control algorithm of trajectory planning, and the algorithm has been successfully validated to plan the path of the airplane’s S-shaped inlet. Qu [10] also proposed a path generation method to overcome the contradiction between angle direction and fiber wrinkles on the S-shape inlet surface, and the proposed method can control the angle deviation within 10 degrees and meet the curvature requirements. However, there are still few studies on the effect of carbon fiber ply angle on the mechanical properties of resin matrix composites. Different carbon fiber ply angles will inevitably affect their service properties. Due to the limited deformation of tow, the local steering radius of the placement path must be greater than the minimum steering radius determined by the material properties, otherwise it will produce wrinkles, tears, and other distortions, which will affect the surface quality and result in a decrease in strength as high as 57.7% [22]. This problem is well-described in References [23,24,25,26], which presents the results of both experimental testing and numerical calculations regarding the influence of placement angle on the properties of composite plates and profiles under compression. The results show that the carbon fiber ply angle has a significant effect on the buckling and post-buckling characteristics of the test structure. In order to predict, reduce, or even avoid those defects, the optimal path planning method and its influence on component performance must be understood.

To sum up, different path planning schemes will seriously affect the strength of the parts. Resin-rich areas, voids, wrinkles, and other defects are easy to appear in the plies, and there are few related researches on the comprehensive consideration of path manufacturability and part strength, so it is urgent to develop corresponding path planning methods to control the manufacturing quality of the composite component. On the other hand, the research of other scholars on the initial trajectory design is not sufficient, and the influence of path design on the mechanical properties of composite components is rarely studied. Therefore, the objective of the work described in this paper is to study the effects of different trajectory planning methods on the mechanical properties of components. The research scope includes finite element simulation calculation and experimental tests of the actual structure.

The subsequent sections, herein, are organized as follows. The generation of fixed angle, geodesic, and variable angle path of the conical shell are discussed in the automated fiber placement process and the steering radius as well as direction deviation are used to evaluate the path quality in Section 2. Afterwards, using the idea of discretization, the actual placement angle of each discrete element on the conical shell is calculated by using the self-developed path angle calculation software, and the strength analysis is carried out for the conical shell with different path design methods to find the optimal path design scheme in Section 3. The strength analysis of conical shells manufactured by different trajectory design schemes is carried out through finite element analysis and actual failure tests in Section 4. Finally, conclusions are drawn in Section 5.

## 2. Path Planning for Composites Conical Shell

Composite conical shell structure has been widely used in aircraft, spacecraft, rockets, and missiles, which are frequently subjected to dynamic loads in service [27,28]. However, it is very challenging to design the roller path of composite conical shell components to satisfy both process and structural requirements. In order to further improve the mechanical properties of composite conical shells, a path design method considering multiple constraints needs to be studied.

### 2.1. Numerical Model

**Figure 1:**
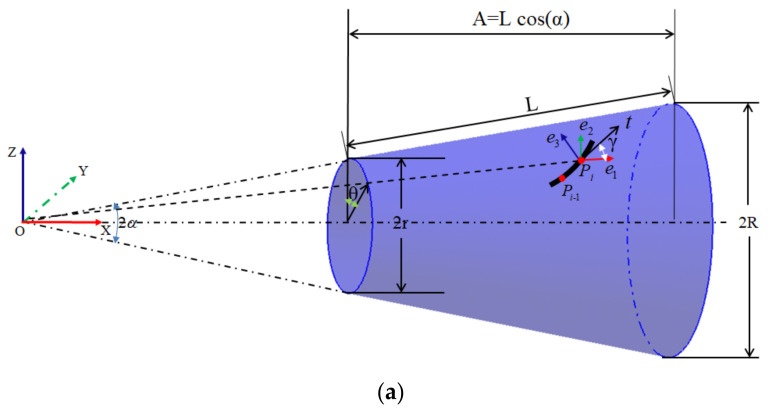

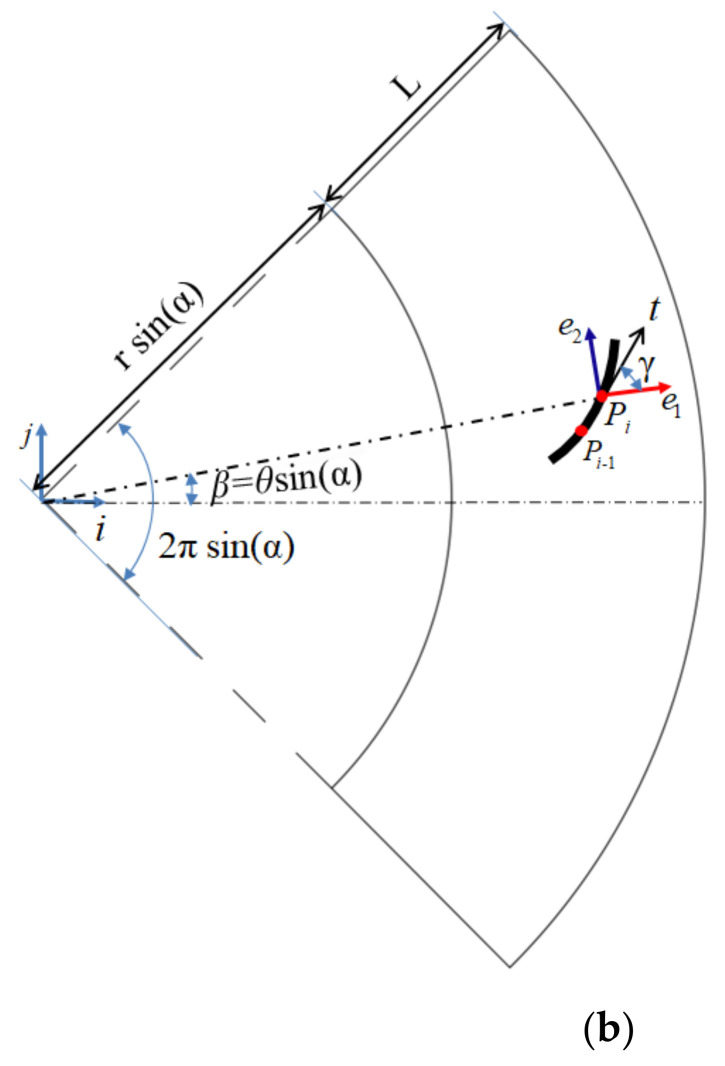
Cone geometry. (**a**) Three-dimensional schematic diagram of the conical shell. (**b**) Developed surface of the conical shell.

A three-dimensional representation of the geometry of a conical shell is shown in Figure 1. In this mathematical model, the basic parameters that are used to define the cone shell are the semi-vertex angle (α), axial length *A* = 1160 mm, smaller radius *r* = 295 mm, and larger radius *R* = 497 mm, and these parameters have the following mathematical relationship:

(1) $$\tan(\alpha )=\frac{R-r}{A}$$

To define the placement angle, the Frenet Frame at $p_{i}$ (*i* is the number of the path) is established, which contains three orthogonal vectors $e_{1}$, $e_{2}$, and $e_{3}$. Here, the unit vectors for the longitudinal and circumferential surface directions, $e_{1}$ and $e_{2}$, respectively, as well as the surface normal $e_{3}$ will be used in the fiber path definitions.

### 2.2. Trajectory of Conical Shell and Its Manufacturability

The composite material can be treated as a material or a structure, whose one outstanding advantage is the designability of performance. Therefore, composite components with different properties can be obtained by different ply directions and ply forms [29]. The quality of path planning directly affects both the quality of offset paths and the mechanical properties of the components. At present, the commonly used path planning methods mainly include fixed angle, geodesic, and variable angle methods [30]. The fixed angle algorithm meets the requirements of structural design, but it is easy to produce wrinkles [31,32]. Compared with the fixed angle method, the geodesic method has an infinite curvature radius and no wrinkle will occur. However, it is very easy to cause the angle deviation to exceed its upper limit, which results in great difference between the actual performance of the component and the design value [13,33]. The variable angle path planning method is an ideal solution for the placement of composite conical shells at present because it comprehensively considers the angle deviation and path curvature.

#### 2.2.1. Initial Path Generation

**Figure 2:**
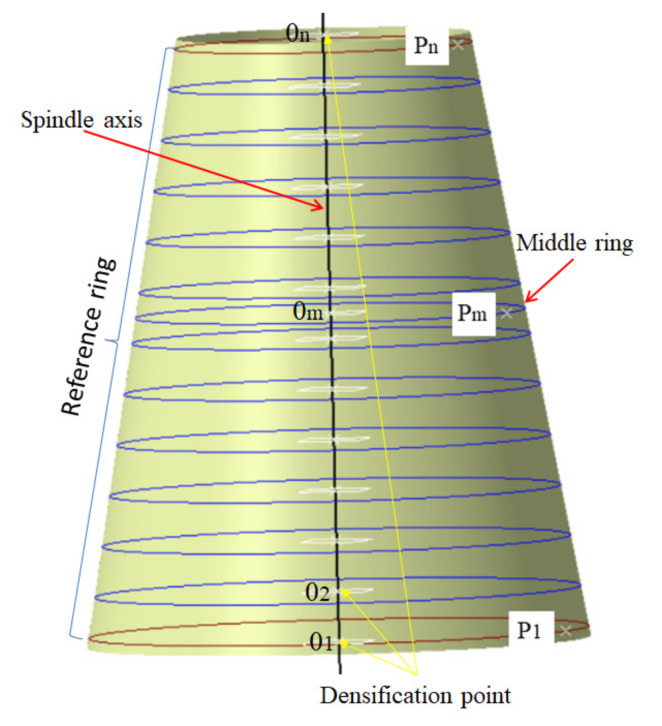
Construction of the initial point.

The starting point is extremely important to the initial path itself and affects the quality of the offset paths. The starting point is obtained according to the following steps in the paper, as shown in Figure 2.

Step 1. The spindle axis is densified into a series of points $O_{i}$ (i = 1, 2, …, n);

Step 2. The slicing ring is formed by the intersection of the mold surface and the normal plane generated by points $O_{i}$ and perpendicular to the spindle axis;

Step 3. The midpoint of the spindle is projected onto the corresponding ring first to form the initial point in Figure 2. If it cannot meet the uniform angle deviation on either side of the initial path, we iterate the points on both sides of the midpoint in turn.

**Figure 3:**
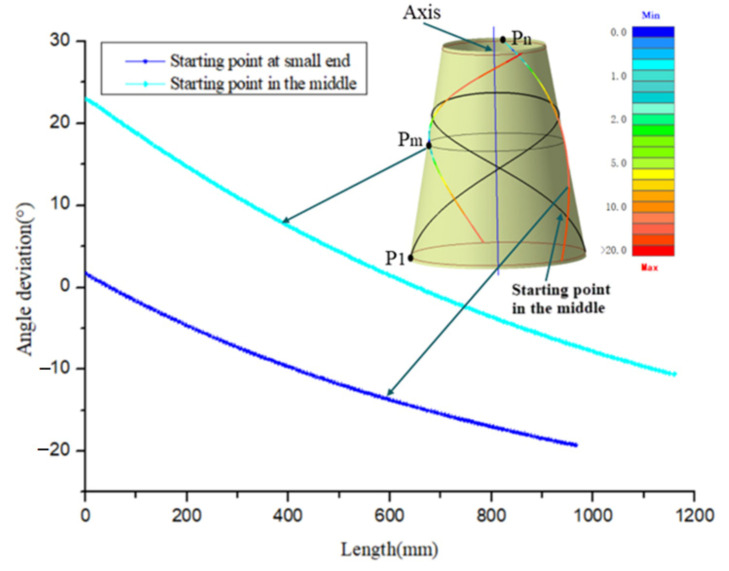
Geodesic trajectory at different starting points.

If the starting point chosen here is not reasonable, it is extremely easy to cause an angle deviation over its upper limit, and cannot generate the required path. Figure 3 shows the geodesic paths at different starting points and their angle deviation. From the Figure 3, we can clearly see that the geodesic path with the starting point P1 (the big end) fails to be generated due to the occurrence of path turn back and unable to reach the boundary of the small end. In addition, even if the angle deviations of the other two geodesic paths are all over the upper limit, their variation laws are completely different.

**Figure 4:**
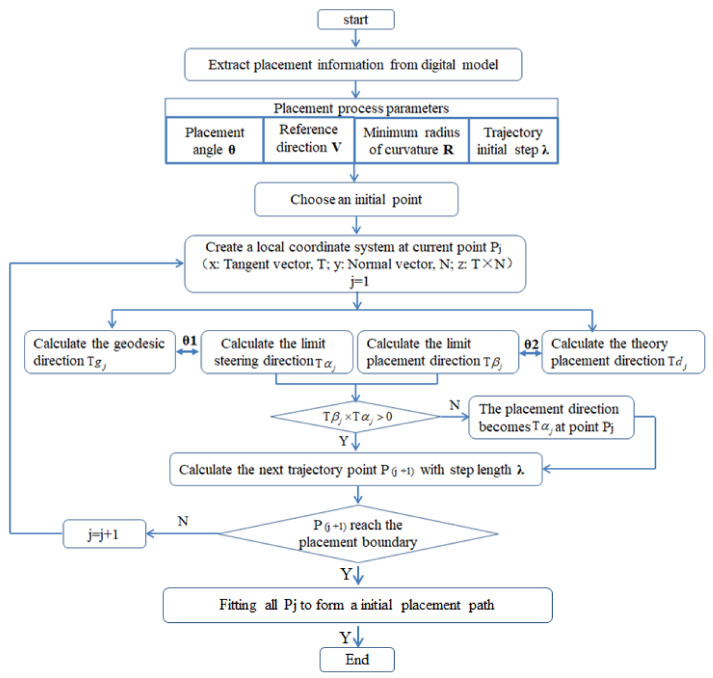
Flow chart of the path generation method.

After the starting point is determined by iterative optimization, the next step is to design the variable angle path on conical shells surfaces, and discrete path points are calculated with a multi constraint model in local Frenet Frames. The flow chart is shown in Figure 4.

**Figure 5:**
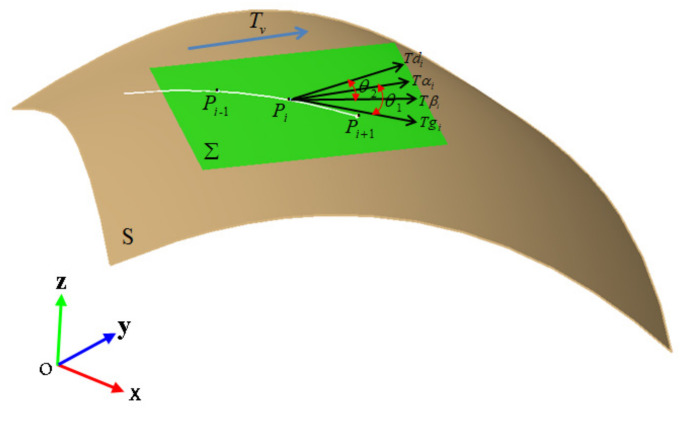
Generation of variable angle trajectory.

As shown in Figure 5, for a given surface S, $T_{v}(X_{v},Y_{v},Z_{v})$ is the reference direction of the fiber path, $P_{i}$ is the path point, and Σ is the tangent plane. All path points are obtained according to the following steps.

Step 1. Select a point $P_{0}$ on the mold surface as the starting point through the above methods;

Step 2. Calculate the normal vector N ($n_{x}{}_{i},n_{y}{}_{i},n_{z}{}_{i}$) of the surface at the points $P_{i}(X_{i},Y_{i},Z_{i})$ and the reference direction $T_{v}$ is projected onto the tangent plane to obtain the projection vector $T\partial_{i}$ at the points $P_{i}$, the coordinates of $T\partial_{i}$ are obtained by the following formula:

(2) $$\left\{\begin{array}{l} X_{\partial }^{\prime }=-\frac{n_{xi}(n_{xi}X_{v}+n_{yi}Y_{v}+n_{zi}Z_{v})}{n_{xi}^{2}+n_{yi}^{2}+n_{zi}^{2}}+X_{i}+\lambda X_{v}\\ Y_{\partial }^{\prime }=-\frac{n_{yi}(n_{xi}X_{v}+n_{yi}Y_{v}+n_{zi}Z_{v})}{n_{xi}^{2}+n_{yi}^{2}+n_{zi}^{2}}+Y_{i}+\lambda Y_{v}\\ Z_{\partial }^{\prime }=-\frac{n_{zi}(n_{xi}X_{v}+n_{yi}Y_{v}+n_{zi}Z_{v})}{n_{xi}^{2}+n_{yi}^{2}+n_{zi}^{2}}+Z_{i}+\lambda Z_{v} \end{array}\right.$$

Step 3. Calculate the $P_{i+1}^{\prime }$ on the tangent plane with a step λ under the three constraint conditions which including angle direction, steering radius and compaction, then $P_{i+1}^{\prime }$ is projected onto the mold surface to get the next path point $P_{i+1}^{}$;

Step 4. Repeat Steps 2 and 3 until the path point reaches the boundary of the surface. All the path points are fitted to form the initial path.

**Figure 6:**
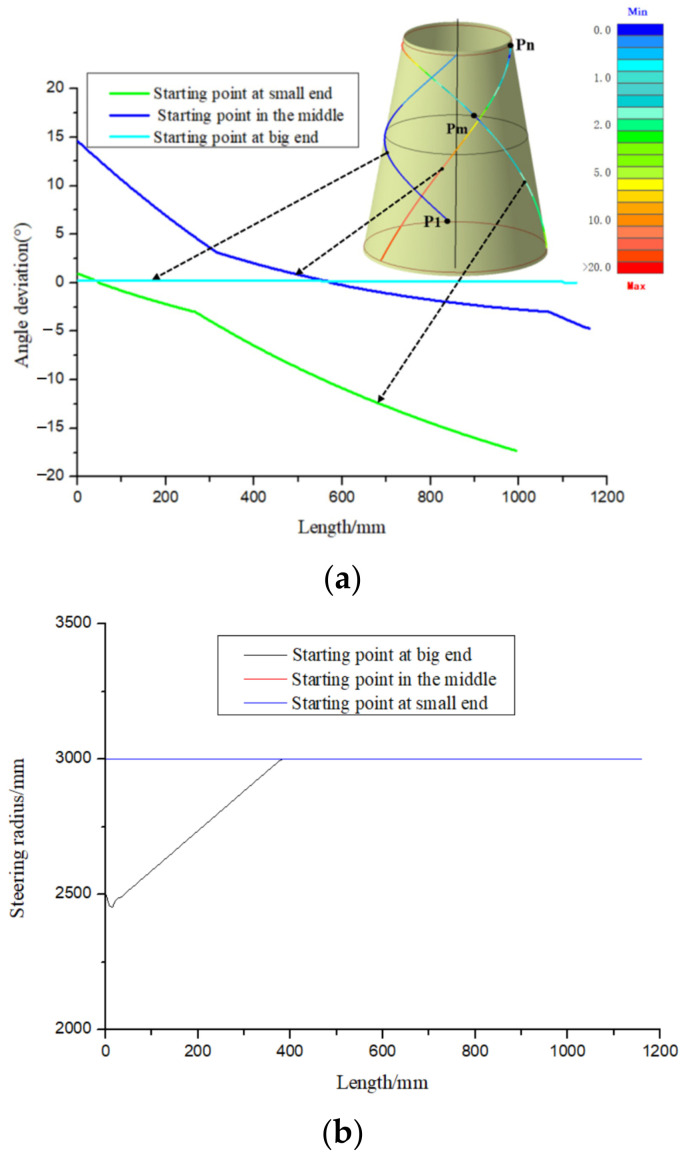
Paths on conical shells surface and its manufacturability. (**a**) Paths and Angle deviation evaluation. (**b**) Fiber steering evaluation.

Using the above method, the 45-degree variable angle path manufacturability of different starting points is analyzed as shown in Figure 6. It can be seen that the variable angle trajectory with the starting point P1 (at the big end) has a good effect on this conical shells surface. The angle deviation is less than 1 degree and the minimum steering radius is greater than 2000 mm. Therefore, point Pm is selected as the starting point of the 45-degree variable angle trajectory in this paper. The initial trajectory design principle of other angles is the same as 45 degrees.

#### 2.2.2. Densification and Coverage Algorithm of the Initial Path

**Figure 7:**
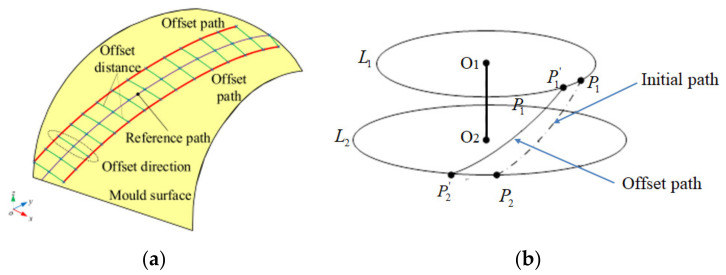
Concept for offsetting the initial path. (**a**) Equidistant offset [7], (**b**) Offset proportionally based on profile perimeter.

To achieve uniform paths over a mold, parallel equidistant offset algorithm is a common method to densify the path for automated fiber placement path planning. It has good applicability for open free curved surfaces with small and medium curvature. However, if the initial path is always offset for the revolving body, the curvature and angle deviation of the offset path will exceed its limit and produce a larger triangle area. It will produce tows breaking enrichment areas and affect the mechanical properties of components. In order to avoid a series of problems existing in the traditional isometric offset path planning algorithm on the rotating body, the offset paths generation algorithm based on partition and circumferential averaging planning algorithm is adopted in this paper, as shown in Figure 7.

**Figure 8:**
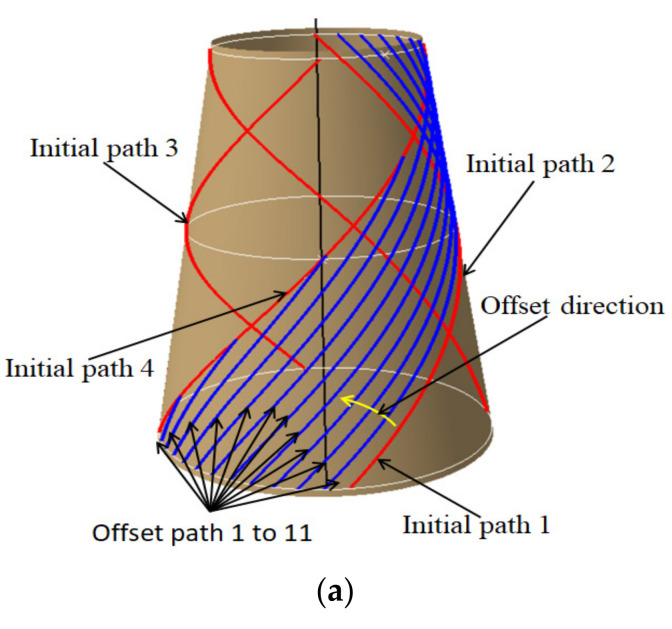

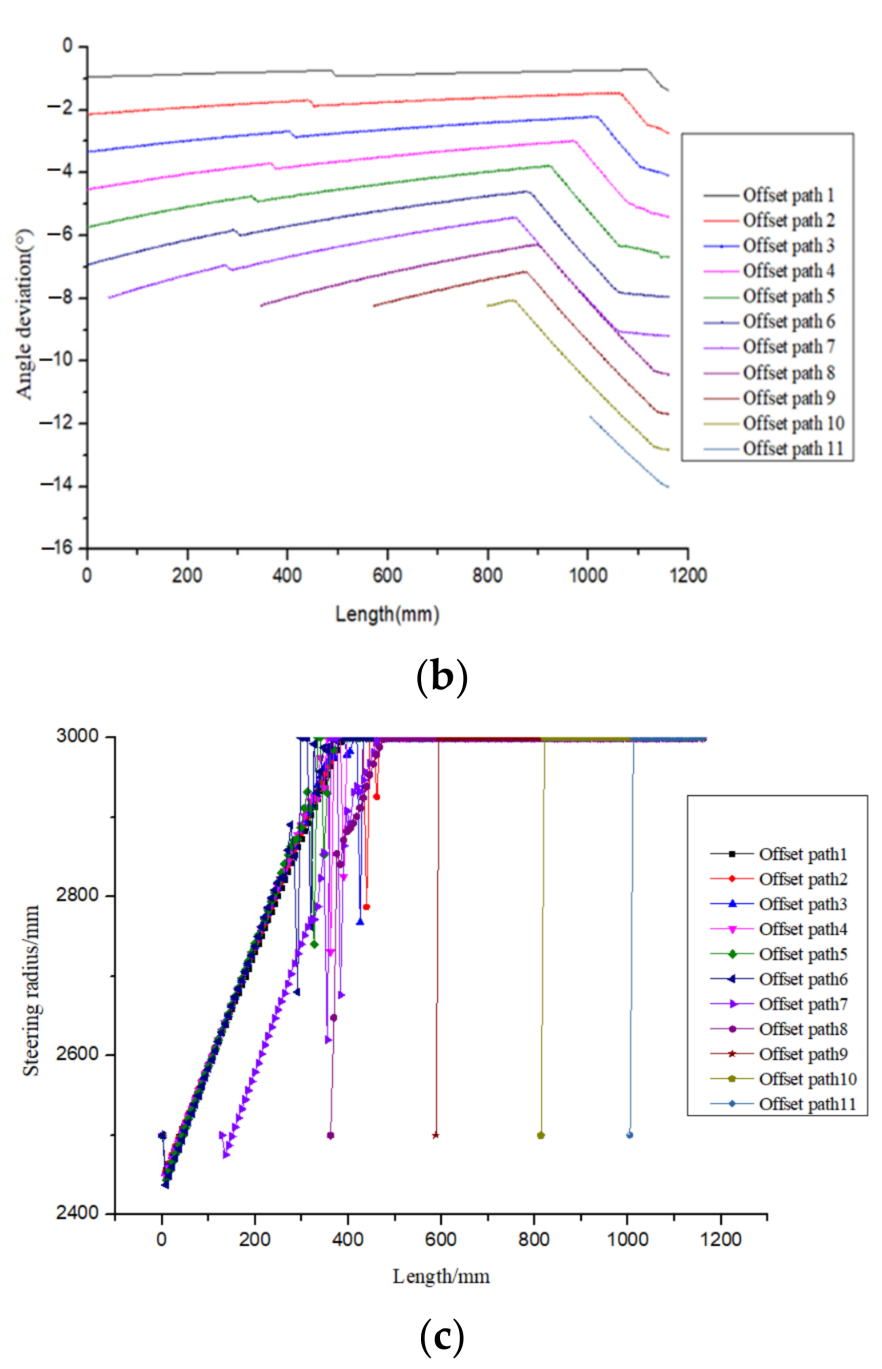
Equidistant offset paths based on partition and its manufacturability. (**a**) Equidistant offset paths based on partition. (**b**) Angular deviation of each offset path. (**c**) Steering radius of each offset path.

**Figure 9:**
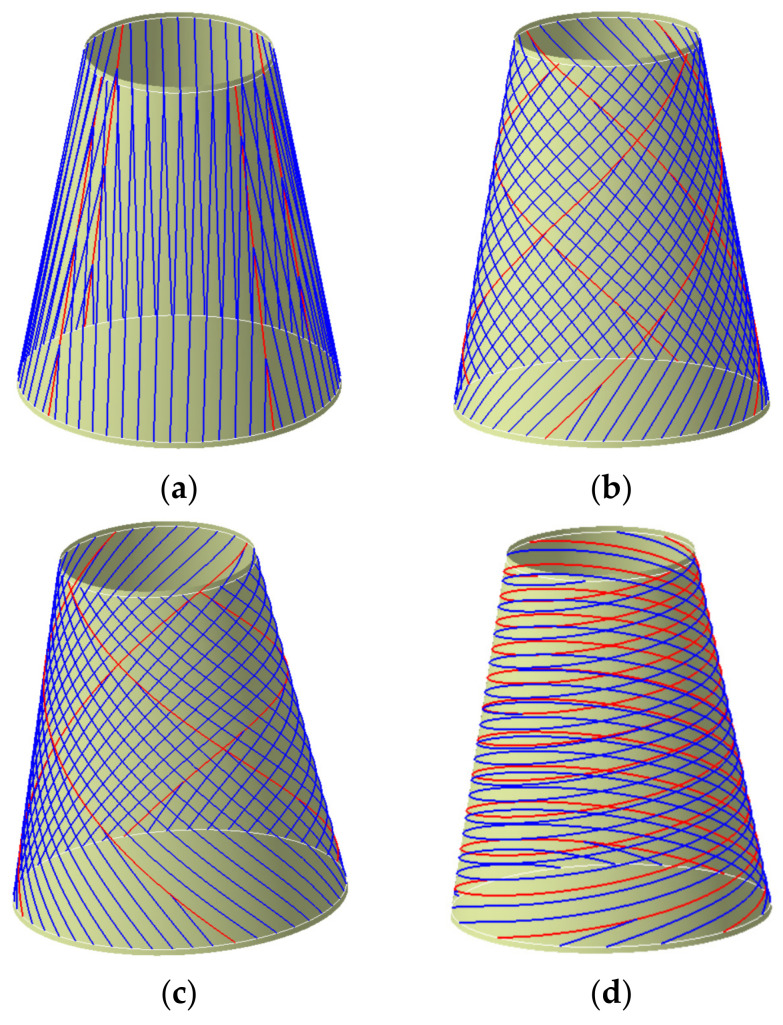
Four typical angle plies are based on partition. (**a**) 0° Ply. (**b**) 45° Ply. (**c**) −45° Ply. (**d**) 80° Ply.

As can be seen from Figure 7a, equidistant offset path is that the reference path is offset by a certain distance (tows number multiplied by tow width) along the direction of the bi-normal vector, whose purpose is to formulate a set of paths without gaps and overlaps between subsequent paths. To avoid the transition concentration of triangular region, the conical shell surface is partitioned in this paper, and the number of partitions depends on the quality of offset paths. In this paper, the conical shell surface is divided into the same four regions according to the variable angle trajectory, and other paths are generated by equidistant offset in each region. Those offset paths and its manufacturability evaluations are shown in Figure 8. The minimum steering radius of offset paths all exceeds 2000 mm and the angle deviation ranges from 0 to 15°. It can be seen that the method also has a good effect on the conical shell surface. Other paths are generated using the same method and Figure 9 shows the laying paths of four angles, the red line is the initial path, and the blue line is the offset path.

**Figure 10:**
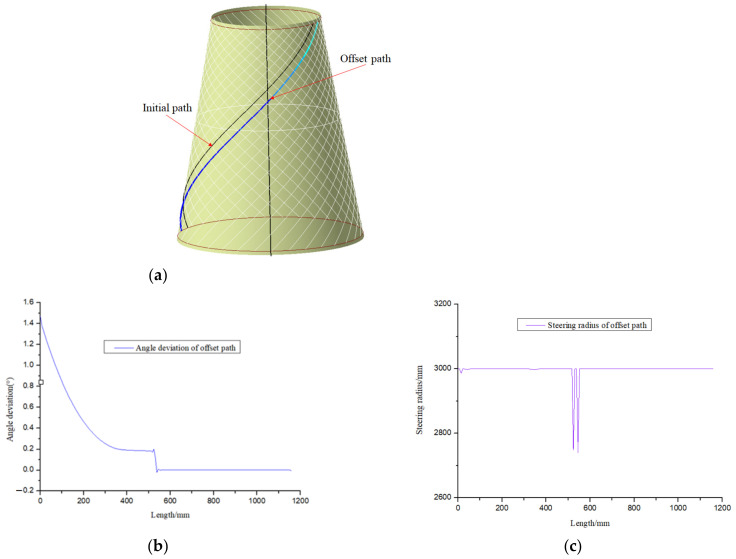
Circumferential averaging planning trajectory and its manufacturability. (**a**) Circumferential averaging path planning. (**b**) Angular deviation of each offset path. (**c**) Steering radius of each offset path.

**Figure 11:**
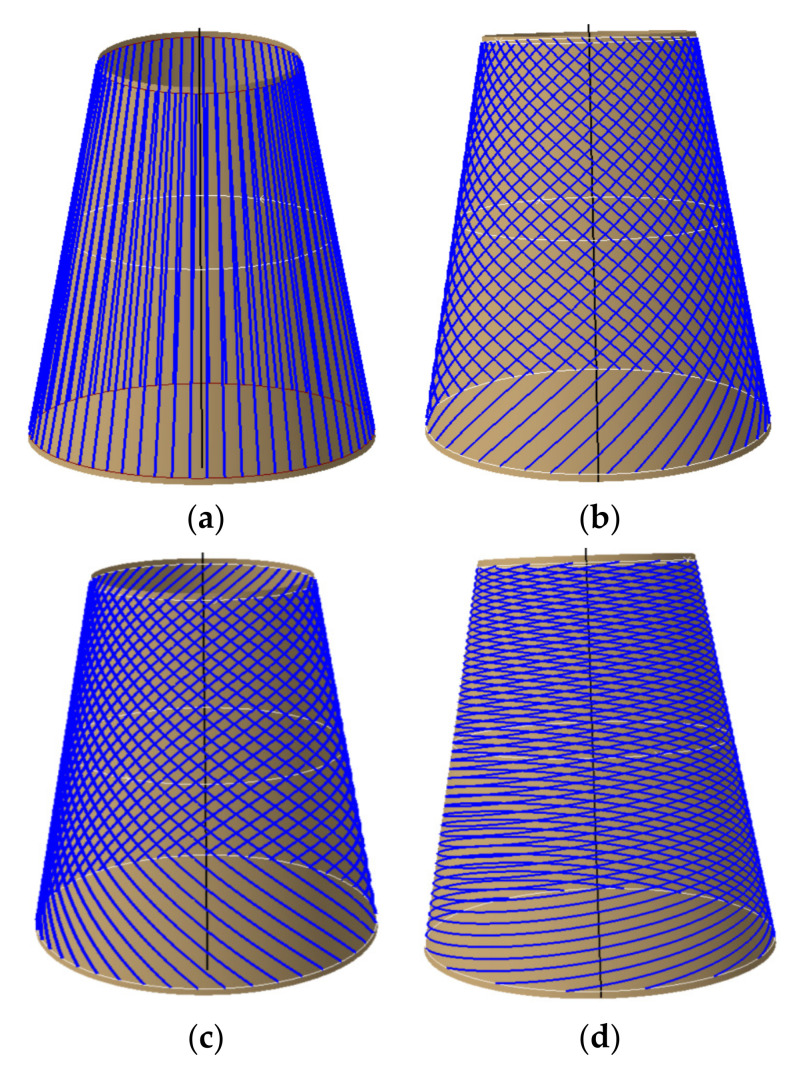
Four typical angle plies based on circumferential averaging. (**a**) 0° Ply. (**b**) 45° Ply. (**c**) −45° Ply. (**d**) 80° Ply.

Different from the equidistant offset paths based on the partition, the placement quality of each circumferential averaging path of the conical shell is the same, and the maximum number of the whole tows is guaranteed. However, it may not meet the technological requirements, which are closely related to the parameters of the cone. Figure 10 shows the circumferential averaging paths based on variable angle and analyzes their manufacturability. From the analysis results, it can be clearly known that the angle deviation of all offset paths is less than 2 degrees, and the minimum steering radius is greater than 2000 mm. Other paths are generated using the same method and Figure 11 shows the laying paths of four angles.

## 3. Finite Element Analysis Based on Path Design

From the previous section, we know that there are different path planning methods for the same conical shell surface, and they all meet the technical requirements. However, how to choose the final path planning method to complete the laying still needs to be compared in terms of strength. According to the ply angle for each element data of typical parts, the strength analysis results are obtained by using ABAQUS simulation software, and compared with the traditional ply angle design in ABAQUS software in this paper.

**Figure 12:**
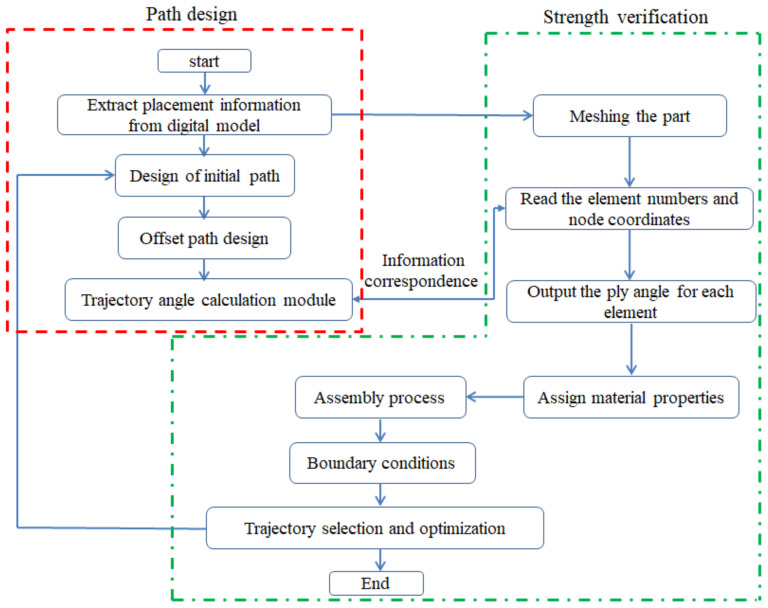
Flow chart of finite element analysis based on trajectory.

In order to realize finite element analysis based on path design, the path design software is developed by CATIA secondary development technology. The software can design the path of fixed angle, geodesic and variable angle, and can calculate the angle of the center point of each triangular patch. The flow chart in Figure 12 illustrates the detailed process.

### 3.1. Material Parameters and Ply Settings

**Figure 13:**
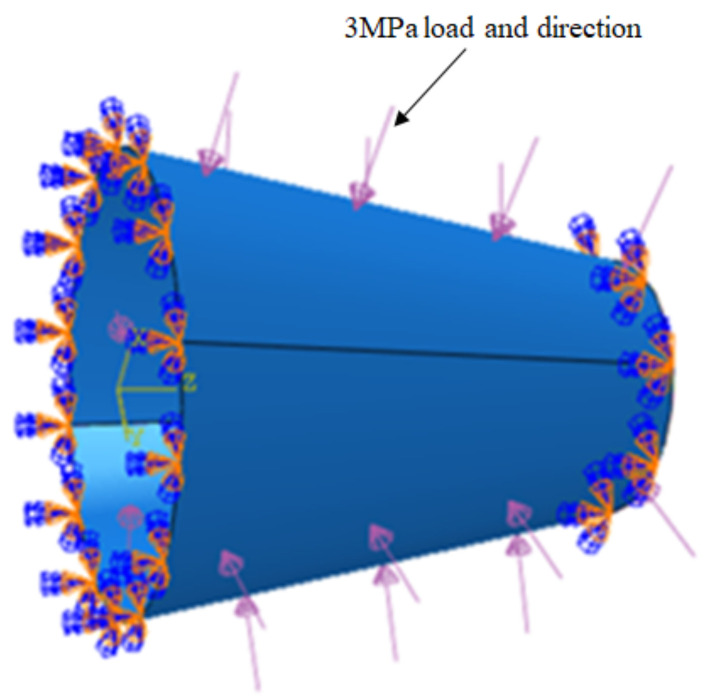
Loading and boundary conditions.

The composite prepreg used in the experiment is EH104-HF40-D6/12 made by Jiangsu Hengshen Co., Ltd. of CHINA, and the performance parameters of the material are shown in Table 1. The prepreg tow had a slitting width of 6.35 mm, a nominal thickness of 0.15 mm. In this paper, the conical shell skin adopts the shell element and S3 element. The total number of units is 8528 and the number of nodes is 4368. The layer of the cone shell model created is symmetrical with 24 layers, the total thickness is 3.6 mm, and the layering sequence is designed as [45/0/−45/90]_3s_. The internal pressure of 3 MPa provided by the design department was applied to the external wall of the conical shell to analyze its static mechanical properties. Static pressure loading is shown in Figure 13. In addition, both the big end and the small end are imposed fixed support constraints.

### 3.2. Simulation Results of Stress Distribution of Composite Conical Shell

The material properties are set according to the same material mechanical properties and the same layup sequence and the same load is applied to analyze the cone shells with two different path design methods (Figure 9 and Figure 11). Table 2 shows the finite element analysis results of the cone shell with two trajectory planning methods of partition equidistant offset and circumferential averaging, which are simulated according to the steps described in Figure 12. From the Table 2, we can clearly see that the distribution of the total displacement nephogram U, fiber transverse stress nephogram S11 and fiber longitudinal stress nephogram S22 of the conical shell under loading, as well as their maximum values as shown in Table 3.

As shown in Table 2 and Table 3, different path design schemes based on variable angle affect not only the displacement of the cone shells, but also the stress distribution. Compared with fixed angle trajectory conical shell, the maximum deformation of circumferential averaging variable angle path conical shell increased by 3.01%, the fiber transverse stress decreased by 6.97%, and fiber longitudinal stress increased by 4.42%. If partition variable angle path is applied, these values can be changed to 23.02, 13.62, and 30.13%, respectively. Therefore, the comprehensive evaluation of the performance of conical shells designed by circumferential averaging variable angle trajectory design scheme is better than the partition variable angle path design scheme.

**Figure 14:**
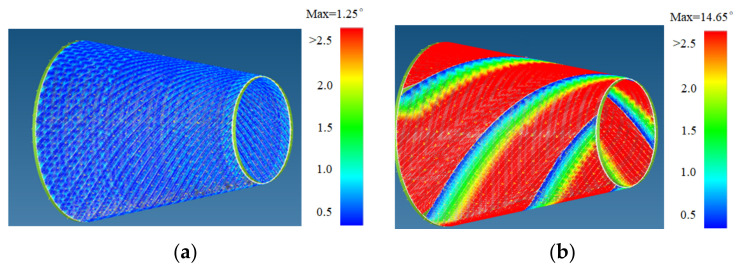
A 45-degree path angle deviation and gaps/overlaps distribution of different path design schemes. (**a**) Circumferential averaging paths. (**b**) Equidistant offset paths based on partition.

Based on the results presented above, the possible explanation is that the size of path angle deviation and the position of the overlapping area formed by different path planning methods have a great impact on the mechanical performance. By comparing the circumferential averaging variable angle path design method (Figure 14a) with partition variable angle path design method (Figure 14b) has smaller angle deviation, and the gaps/overlaps position are more evenly distributed on the surface of the conical shell. Therefore, the organic combination of defect suppression, path planning, and strength analysis greatly ensures the rationality of trajectory design, which makes it possible to lay high-quality and efficiency, especially for complex rotating bodies.

## 4. Experimental and Analysis

**Figure 15:**
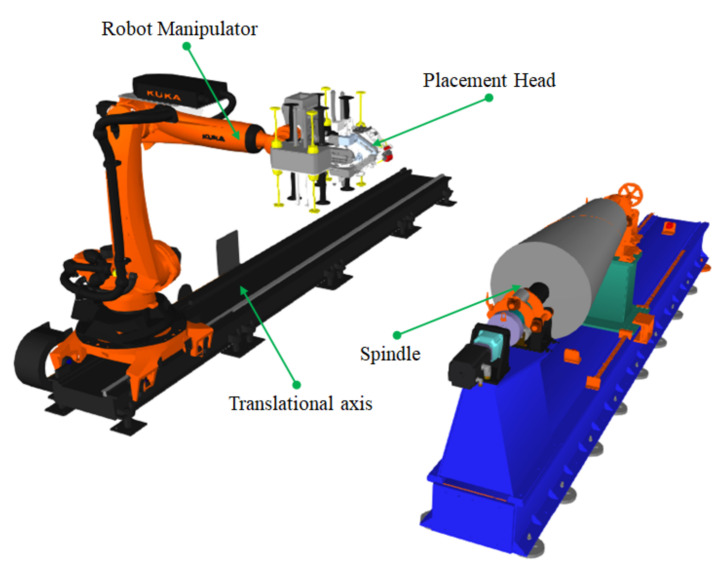
Robot automatic fiber placement platform.

To verify the feasibility of the above scheme, the placement experiment of the composite conical shell described in the previous paper is completed by using the circumferential averaging paths and equipment developed independently by our research team. As shown in Figure 15, the automatic fiber placement platform includes fiber placement head, KUKA 6-DOF robot, translation guiderail and spindle, and the guiderail can move the platform in the Y direction and greatly increase the movement capacity in the axis direction of the spindle. The equipment can realize flying cutting/flying sending operation at the laying speed greater than 200 mm/s and can lay up to 8 tows with the width of a single tow is 6.35 mm, simultaneously. Moreover, the prepreg used for laying is EH104-HF40-D6/12, and its corresponding minimum turning radius was 1500 mm which was measured by experiment under the conditions of our common laying technology. Under the process conditions of laying temperature of 35 °C, laying speed of 150 mm/s, belt tension of 4 N and laying forces of 500 N, the laying experiment was carried out with the fiber placement platform.

**Figure 16:**
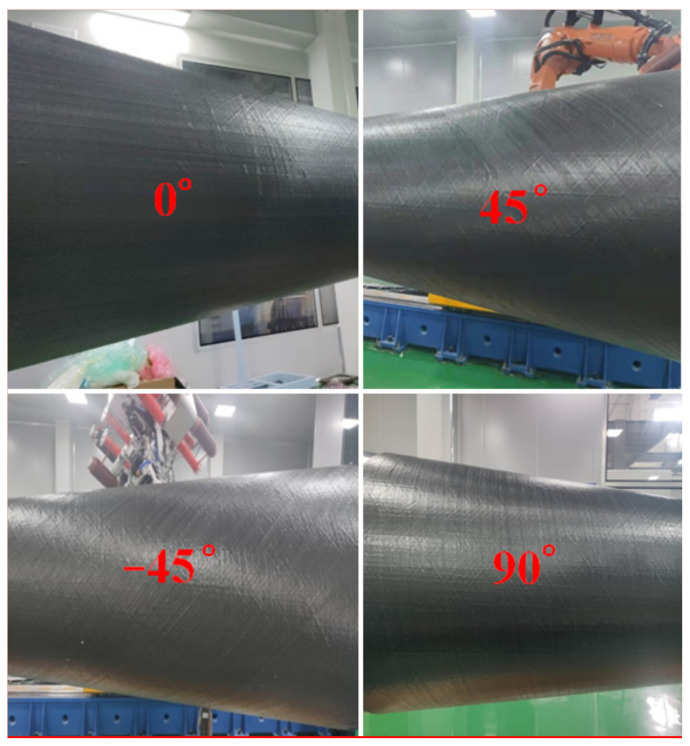
The placement experiment results on the conical shell surface.

The placement experiment results of four typical plies are shown in Figure 16. It can be seen that the tow fits well with the conical shell surface, and there are neither visible wrinkles nor gaps and overlaps in each ply. This fully shows that the path generation method has strong engineering practicability and can well ensure the quality of tow placements. Therefore, through the path design and optimization based on the laying process and the mechanical properties of the parts, it greatly meets the strength requirements of the part while ensuring the quality of the path laying, and also improves the efficiency of path planning.

**Figure 17:**
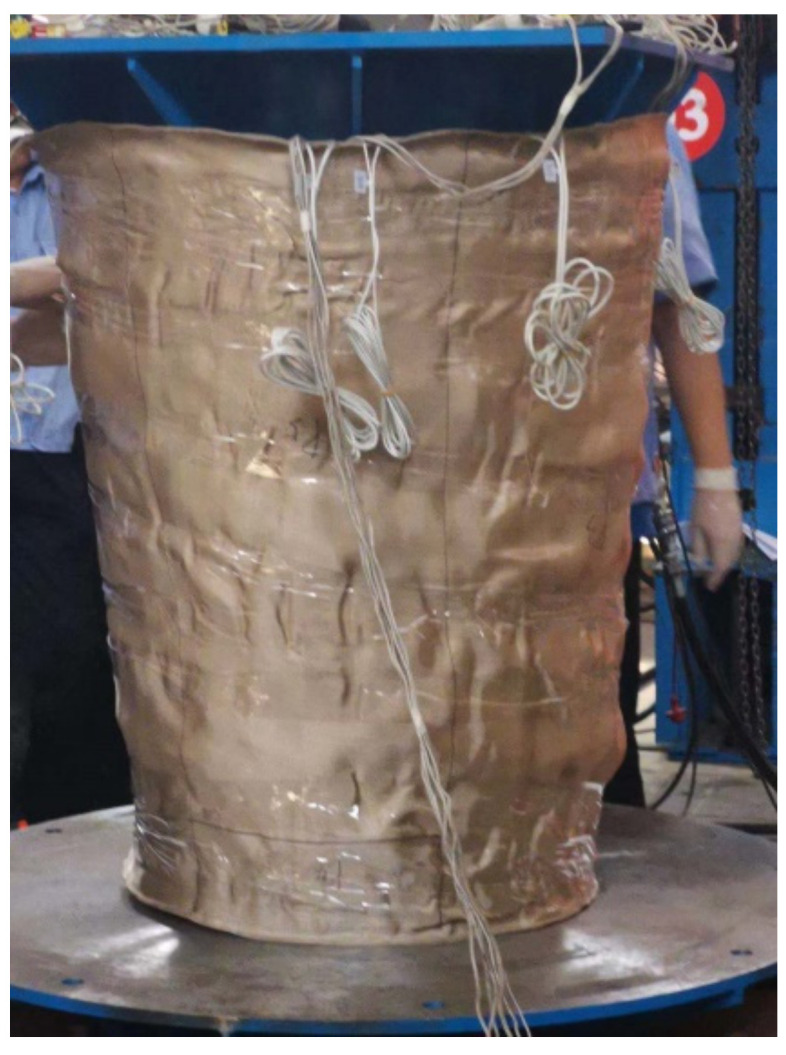
Mechanical properties test on the conical shell surface.

**Figure 18:**
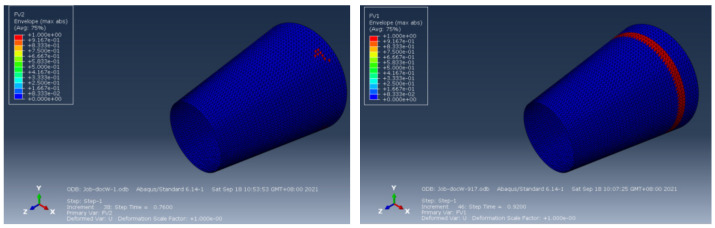
Mechanical properties test on the conical shell surface.

In order to verify that the optimized placement path has good quality, the strength of composite components manufactured by two different path design schemes is analyzed. The experimental tests (Figure 17) were conducted in the full range of loading, until the structure’s failure. In addition, numerical simulations were conducted the finite element method in ABAQUS, as shown in Figure 18. The total load is 6 MPa. The set load increased by 6/50 for each incremental step, which is reflected in the time step. Increase the time of each incremental step by 1/50 = 0.02 s. The experimental results and simulation analysis are shown in Table 4.

From Table 4, we get the following conclusions. The simulation results show that the conical shell manufactured by the partition variable angle path is damaged when the load increases to 4.56 MPa, and the experimental test result is 4.32 MPa. The experimental and simulation results are basically consistent. If the circumferential uniform variation angle path is applied, these values can be changed to 5.52 and 5.38 MPa, respectively. Therefore, both experiments and simulation show that the design of circumferential uniform variation angle path has good quality, as well as proves the rationality of the trajectory design method proposed in this paper. The path planning algorithm that coordinates path planning and defect suppression play an important role in optimizing placement trajectory and improving mechanical properties of parts.

## 5. Conclusions

The placement path design of conical shell surfaces mainly includes the generation of initial trajectory and offset trajectory. Different path design methods have different mechanical properties of components, and they are very different. In order to optimize and select the optimal placement path, the path planning algorithm of coordinated path planning and defect suppression is studied. The conclusions can be summarized in the following point.

(1) The initial point is extremely important to the initial path and offset paths. If the initial point selection is unreasonable, on the one hand, which will lead to the failure of path generation because the occurrence of path turns back and cannot reach the boundary, on the other hand, when taking the initial point close to the boundary, the angle deviation can reach more than 20°, which cannot meet placement requirement. Therefore, it is necessary to iteratively optimize the initial point to design the initial path to make it have the best laying quality.
(2) The fixed angle algorithm meets the requirements of structural design, but it is easy to produce wrinkles. Compared with the fixed angle method, the geodesic method has an infinite curvature radius and no wrinkles will occur. However, it is very easy to cause the angle deviation to exceed its upper limit. The variable angle trajectory planning method is an ideal solution for the placement of composite conical shells at present because it comprehensively considers the angle deviation and steering radius of the path. For this cone shell, the angle deviation of the variable angle path designed by the path design software developed independently by our research group is less than 2 degrees, and the minimum steering radius of the path is greater than 2000 mm.
(3) By comparing the circumferential averaging variable angle path design method (Figure 13a), the partition variable angle path design method (Figure 14b) has smaller angle deviation, and the gaps/overlaps position are more evenly distributed on the surface of the conical shell. The possible explanation is that the size of path angle deviation and the position of the overlapping area formed by different path planning methods have a great impact on the mechanical performance. Therefore, the organic combination of defect suppression, path planning, and strength analysis greatly ensures the rationality of path design, which makes it possible to lay high-quality and efficient, especially for complex rotating bodies.
(4) According to the finite element analysis results of the cone shell with two trajectory planning methods of partition variable angle path and circumferential averaging variable angle path, it can be observed that the maximum deformation and maximum transverse and longitudinal stress of fiber of circumferential averaging variable angle path conical shell are reduced by 16.3%, 5.85%, and 19.76%, respectively, than that of the partition variable angle trajectory. Considering the manufacturability and strength of all laying paths, the design scheme based on circumferential averaging variable angle path is better than the partition variable angle path design scheme.
(5) The actual placement experiment proves that the proposed path generation method can obtain a good placement effect. This fully shows that the path generation method has strong engineering practicability and can well ensure the quality of tow placement. In addition, both experiments and Finite element simulation results further verify the rationality of path design method proposed in this paper. The path planning algorithm that coordinates path planning and defect suppression plays an important role in optimizing placement trajectory and improving mechanical properties of parts.

## Figures and Tables

**Table 1:** Basic mechanical property parameters of materials.

Property	E_1_/GPa	E_2_/GPa	G_12_/GPa	G_13_/GPa	G_23_/GPa	$v_{12}$
Values	170	8.6	4.8	4.8	3.4	0.2823

**Table 2:** Nephogram of displacement and stress distribution.

Fixed Angle Path	Partition Variable Angle Path	Circumferential Averaging Variable Angle Path
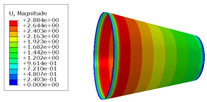	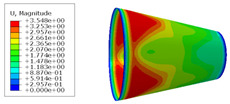	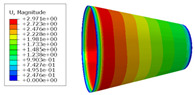
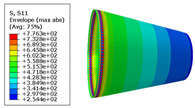	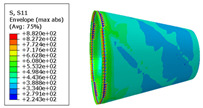	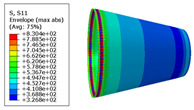
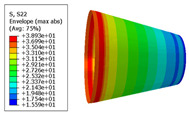	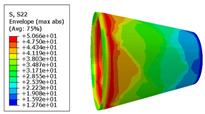	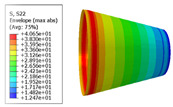

**Table 3:** Maximum displacement and maximum stress of different path design schemes.

Trajectory Design Scheme	U Max (mm)	S11 Max (MPa)	S22 Max (MPa)
Fixed angle trajectory	2.884	776.3	38.93
Partition variable angle trajectory	3.548	882.0	50.66
Circumferential averaging variable angle trajectory	2.971	830.4	40.65

**Table 4:** Results of both experimental testing and numerical calculations.

Trajectory Design Scheme	Finite Element Simulation	Experimental Test
Partition variable angle trajectory	4.56 MPa	4.32 MPa
Circumferential averaging variable angle trajectory	5.52 MPa	5.38 MPa

## Data Availability

All data, models, or code that support the conclusions of this study are available from the corresponding author upon reasonable request.

## References

[1] Shirinzadeh B., Aized T. (2011). Robotic fiber placement process analysis and optimization using response surface method. Int. J. Adv. Manuf. Technol..

[2] Wen L., Xiao J., Wang X. (2015). Research progress of automatic composite placement technology in China. J. Nanjing Univ. Aeronaut..

[3] Asif M., Ahmed I. (2017). Advanced composite material for aerospace application—A review. Int. J. Eng. Manuf. Sci..

[4] Marsh G. (2011). Automating aerospace composites production with fiber placement. Reinf. Plast..

[5] Lemaire E., Zein S., Bruyneel M. (2015). Optimization of composite structures with curved fiber trajectories. Compos. Struct..

[6] Zhao C., Xiao J., Li Y., Chu Q., Xu T., Wang B. (2017). An Experimental Study of the Influence of in-Plane Fiber Waviness on Unidirectional Laminates Tensile Properties. Appl. Compos. Mater..

[7] Long Y., Chen Z., Shi Y., Mo R. (2014). An accurate approach to roller path generation for robotic fiber placement of free-form surface composites. Robot. Comput. Integr. Manuf..

[8] Schueler K., Miller J., Hale R. (2004). Approximate geometric methods in application to the modeling of fiber placed composite structures. J. Comput. Inf. Sci. Eng..

[9] Duan Y., Ge Y., Xin Z. (2015). Trajectory planning of fiber placement based on controlled angle and interval. J. Aeronaut..

[10] Qu W., Gao J., Yang D., He R., Yang Q., Cheng L., Ke Y. (2021). Automated fiber placement path generation method based on prospective analysis of path performance under multiple constraints. Compos. Struct..

[11] Shirinzadeh B., Cassidy G., Oetomo D., Alici G., Ang M. (2007). Trajectory generation for open-contoured structures in robotic fiber placement. Robot. Comput. Integr. Manuf..

[12] Sonmez F., Akbulut M. (2007). Process optimization of tape placement for thermoplastic composites. Compos. A.

[13] Brooks T., Martins J. (2018). On manufacturing constraints for tow-steered composite design optimization. Compos. Struct..

[14] Falcóa O., Mayugo J., Lopes C., Gascons N., Turon A., Costa J. (2014). Variable-stiffness composite panels: As-manufactured modeling and its influence on the failure behavior. Compos. Part B Eng..

[15] Gao J., Qu W., Yang D., Zhu W., Ke Y. (2021). Two-Stage Sector Partition Path Planning Method for Automated Fiber Placement on Complex Surfaces. Comput. Aided Des..

[16] Zhao C., Xiao J., Huang W., Huang X., Gu S. (2016). Layup quality evaluation of fiber trajectory based on prepreg tow deformability for automated fiber placement. J. Reinf. Plast Compos..

[17] Zhang P., Sun R., Zhao X., Hu L. (2015). Placement suitability criteria of composite tape for mould surface in automated tape placement. Chin. J. Aeronaut..

[18] Qu W., He R., Cheng L., Yang D., Gao J., Wang H., Yang Q., Ke Y. (2021). Placement suitability analysis of automated fiber placement on curved surfaces considering the influence of prepreg tow, roller and AFP machine. Compos. Struct..

[19] Blom A., Stickler P., Gürdal Z. (2010). Optimization of a composite cylinder under bending by tailoring stiffness properties in circumferential direction. Compos. Part B.

[20] Xiong W., Xiao J., Wang X., Li J., Huang Z. (2013). Algorithm of Adaptive Path Planning for automated Placement on Meshed Surface. Acta Aeronaut. Et Astronaut. Sin..

[21] Shirinzadeh B., Alici G., Foong C., Cassidy G. (2004). Fabrication process of open surfaces by robotic fiber placement. Robot. Comput. Integr. Manuf..

[22] Zhao C., Wang B., Xiao J. (2016). Macroscopic characterization of fibermicro-buckling and its influence on composites tensile performance. J. Reinf. Plast. Compos..

[23] Debski H., Rozylo P., Teter A. (2021). Buckling and limit states of thin-walled composite columns under eccentric load. Thin Walled Struct..

[24] Zimmermann N., Wang P.H. (2020). A Review of Failure Modes and Fracture Analysis of Aircraft Composite Materials. Eng. Fail. Anal..

[25] Rozylo P., Debski H., Wysmulski P., Falkowicz K. (2018). Numerical and experimental failure analysis of thin-walled composite columns with a top-hat cross section under axial compression. Compos. Struct..

[26] Wu K., Turpin J., Stanford B. Structural Performance of Advanced Composite tow-steered shells with cutouts. Proceedings of the 55th AIAA/ASME/ASCE/AHS/ASC Structures, Structural Dynamics, and Materials Conference.

[27] Hu H., Ou S. (2001). Maximization of the fundamental frequencies of laminated truncated conical shells with respect to fiber orientations. Compos. Struct..

[28] Blom A., Tatting B., Hol J., Guerdal Z. (2009). Fiber path definitions for elastically tailored conical shells. Compos. Part B.

[29] Beakou A., Cano M., Cam J., Verney V. (2011). Modelling slit tape buckling during automated prepreg manufacturing: A local approach. Compos. Struct..

[30] Duvaut G., Terrel G., Lene F. (2000). Optimization of fiber reinforced composites. Compos. Struct..

[31] Ghiasi H., Fayazbakhsh K., Pasini D., Lessard L. (2010). Optimum stacking sequence design of composite materials Part II: Variable stiffness design. Compos. Struct..

[32] Wehbe R., Tatting B., Rajan S., Harik R., Sutton M., Gürdal Z. (2020). Geometrical modeling of tow wrinkles in automated fiber placement. Compos. Struct..

[33] Shinya H., Teruki I., Narita Y. (2013). Multi-objective optimization of curvilinear fiber shapes for laminated composite plates by using NSGA-II. Compos. Part B Eng..

